# Electrical impedance tomography for predicting failure of spontaneous breathing trials in patients with prolonged weaning

**DOI:** 10.1186/s13054-017-1758-2

**Published:** 2017-07-12

**Authors:** Johannes Bickenbach, Michael Czaplik, Mareike Polier, Gernot Marx, Nikolaus Marx, Michael Dreher

**Affiliations:** 1 Department of Intensive Care Medicine, University Hospital of the RWTH Aachen, Pauwelsstraße 30, 52074 Aachen, Germany; 2 Department of Anesthesiology, University Hospital RWTH Aachen, Aachen, Germany; 3 Department of Cardiology, Pneumology, Angiology and Intensive Care Medicine, University Hospital RWTH Aachen, Aachen, Germany

**Keywords:** Mechanical ventilation, Ventilatory heterogeneity, Electrical impedance, Tomography, Prolonged weaning

## Abstract

**Background:**

Spontaneous breathing trials (SBTs) on a T-piece can be difficult in patients with prolonged weaning because of remaining de-recruitment phenomena and/or insufficient ventilation. There is no clinically established method existent other than experience for estimating whether an SBT is most probably beneficial. Electrical impedance tomography (EIT) is a clinical useful online monitoring technique during mechanical ventilation, particularly because it enables analysis of effects of regional ventilation distribution. The aim of our observational study was to examine if EIT can predict whether patients with prolonged weaning will benefit from a planned SBT.

**Methods:**

Thirty-one patients were examined. Blood gas analysis, vital parameter measurements, and EIT recordings were performed at three time points: (1) baseline with pressure support ventilation (PSV) (t0), (2) during a T-piece trial (t1), and (3) after resumption of PSV (t2). Calculation of EIT parameters was performed, including the impedance ratio (IR), the tidal variation of impedance (TIV), the changes in end-expiratory lung impedance (ΔEELI), the global inhomogeneity index (GI), and the regional ventilation delay (RVD) index with use of different thresholds of the percentage inspiration time (RVD40, RVD60, RVD80). The predictive power of the baseline GI with regard to clinical impairment of an SBT was analyzed by means of ROC curves. Clinical deterioration was assumed when tidal volume was decreased by at least 20 ml after the T-piece trial, measured at t2.

**Results:**

Partial pressure of arterial oxygen significantly decreased at t1 (71 ± 15 mmHg) compared with t0 (85 ± 17 mmHg, *p* < 0.05) and t2 (82 ± 18 mmHg, *p* < 0.05). The IR trended toward higher values during t1. At t1, TIV and ΔEELI significantly decreased. The GI was significantly increased at t1 (t0 59.3 ± 46.1 vs t1 81.5 ± 62.5, *p* = 0.001), as were all RVD indexes. Assuming a GI cutoff value of >40, sensitivity of 85% and specificity of 50% were reached for predicting an increased future tidal volume.

**Conclusions:**

EIT enables monitoring of regional ventilation distribution during SBTs and is suitable to estimate whether an SBT probably will be beneficial for an individual patient. Therefore, the application of EIT can support clinical decisions regarding patients in the phase of prolonged weaning.

## Background

Electrical impedance tomography (EIT) is an imaging technique that provides images of regional ventilation distribution in the lungs by measuring intrathoracic changes of electrical impedance. Based on application of a small current using electrodes on the skin surface, the measured impedance changes are integrated in a cross-sectional picture showing the amount of air in an analyzed lung region [1]. EIT is increasingly taken into consideration as a diagnostic tool to guide ventilation distribution and end-expiratory lung volume in critically ill patients requiring mechanical ventilation (MV) because of respiratory failure [2, 3]. In diverse clinical studies, the potential of EIT in monitoring settings of positive end-expiratory pressure (PEEP) for analysis of lung recruitability and for avoidance of ventilator-induced lung injury is a particular focus [4–7]. Moreover, other different clinical scenarios, such as one-lung ventilation, lung edema, endotracheal suctioning, or spontaneous breathing, have proven the usability of EIT [8–11].

Prolonged weaning (with an incidence of 15%) from MV [12] defines patients who are in need of a complex and protracted treatment (at least three spontaneous breathing trials [SBTs] or ≥7 days of ventilation) [13] to complete the process of discontinuation from the ventilator. Reasons are their comorbidities (e.g., chronic obstructive pulmonary disease), prolonged treatment in the intensive care unit (ICU) in consequence of acute conditions (e.g., septic or cardiogenic shock, acute respiratory failure), and subsequent severe weakness of respiratory muscles [14], as well as fluid overload. The treatment of this patient group is particularly focused on the weaning process from MV by extensively restoring respiratory workload and muscular conditions. In first T-piece trials, concurrent symptoms of ventilator insufficiency caused by remaining muscular compromise as well as increasing de-recruitment of the lung resulting from still-existing lung edema may interfere with the patients’ SBT. Clinical signs of rapid shallow breathing and respiratory fatigue often lead to termination of SBTs with a T-piece. Because bedside monitoring of changes of end-expiratory lung volume and regional ventilation distribution is normally missing, SBT is usually guided by clinical signs and experience. However, in some cases, patients’ capacities are exceeded very abruptly and unexpectedly. Thus, EIT could be a potential technology for use in monitoring increasing regional inhomogeneities of the lungs and de-recruitment phenomena in real time, and it is especially faster than blood gas analysis or clinical signs. Interestingly, there is no clinical study published regarding the use of EIT in patients with prolonged weaning from MV.

The aim of this observational study was to analyze the usefulness of EIT in SBTs in a patient group with prolonged weaning. Therefore, EIT recordings were performed before, during, and after SBTs in a patient group with prolonged weaning. The particular focus of this study was to examine the predictive power of the global inhomogeneity index (GI) in terms of a decreased tidal volume (V_T_) after performing the SBT using a T-piece.

## Methods

This observational study received approval from the institutional review board for human studies at the University Hospital Aachen, Germany. Patients’ confidentiality was maintained. This study was conducted at the interdisciplinary weaning unit of the University Hospital RWTH Aachen.

### Patients and data collection

During April 2014 and April 2015, patients who were treated on the weaning unit because of prolonged weaning after cardiac surgery or septic/cardiogenic shock were routinely examined by EIT and consecutively enrolled. Patients underwent cardiac surgery (coronary artery bypass graft, valve surgery, or combined procedures) or had an acute shock (cardiogenic or septic) and had a process of long-term ventilation followed by prolonged weaning as defined by Boles et al. (at least three SBTs or ≥7 days of ventilation) [13]. All patients were tracheotomized under pressure support ventilation (PSV) and fulfilled “ready-to-wean” criteria as used in clinical routine:

- Partial pressure of arterial oxygen (PaO_2_) ≥60 mmHg with fraction of inspired oxygen (FiO_2_) ≤0.4
- PEEP ≤8 cmH_2_O
- Glasgow Coma Scale score >13 and/or Richmond Agitation-Sedation Scale 0/−1
- Body temperature <38 °C
- Hemoglobin >80–100 g/L
- Low dosages of vasopressors or sedatives [15]

Data were retrieved from an electronic patient record system (medico//s; Siemens, Munich, Germany) and from an online patient data documentary system (IntelliSpace Critical Care and Anesthesia, ICCA Rev. F.01.01.001; Philips Electronics, Amsterdam, The Netherlands). Data on age, sex, ICU length of stay prior to admission to the weaning unit, and time on MV before admission to the weaning unit were documented.

### Study design and measurements

**Fig. 1:**
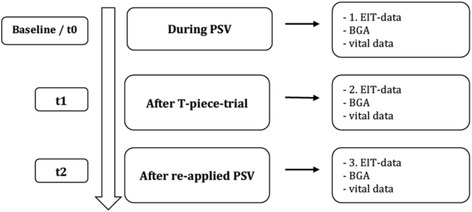
Electrical impedance tomography (EIT) measurements were performed after the protocol during t0, t1, and t2. *BGA* Blood gas analysis, *PSV* Pressure support ventilation

EIT measurements for 15 minutes were executed after a standardized procedure using a PulmoVista 500 (Dräger Medical GmbH, Lübeck, Germany). Three measurement time points were chosen: t0 (as a baseline measurement), t1 (at the end of a 15-minute T-piece trial, resulting in an overall time on the T-piece of at least 30 minutes), and t2 (15 minutes after return on the ventilator with PSV) (Fig. 1). All patients were positioned with the head elevated at 30 to 45 degrees. A 16-electrode belt was used and positioned between the fourth and sixth intercostal space of the thorax. A reference electrode was positioned at the abdominal region. Contact gel was used for adequate skin contact. After calibration, a so-called tidal image was generated with every breath by creating an image of ventilation from the difference between the end and the beginning of each inspiration.

Vital data (including body temperature, heart rate, systolic arterial pressure, mean arterial pressure, diastolic arterial pressure, need for vasopressors, peripheral oxygen saturation, and clinical signs of respiratory fatigue [respiratory rate {RR} and rapid shallow breathing index {RSBI}] at time points t0 and t2 on the ventilator) were continuously monitored and documented at all measured time points. Likewise, ventilation parameters, including fraction of inspired oxygen (FiO_2_), level of PEEP and inspiratory airway pressure (pressure support) as well as PaO_2_, partial pressure of arterial carbon dioxide (PaCO_2_), and further blood gas analysis values, were documented.

### Offline EIT analysis

Functional EIT images were analyzed with the Dräger EIT Data Analysis Tool 6.1 (Dräger Medical GmbH, Lübeck, Germany) and EITdiag v.4 (Dräger Medical GmbH, Lübeck, Germany). A low-pass filter with a cutoff frequency of 50 minute^−1^ was applied to exclude cardiac-related variations.

Within the generated tidal images, four horizontal layers were defined as regions of interest (ROIs) and numbered from 1 to 4 (from ventral to dorsal). The impedance ratio (IR), which is used for determining regional ventilation distribution between dependent (dorsal) and nondependent (ventral) parts of the lungs, was calculated by dividing the sum of ROI1 and ROI2 by the sum of ROI3 and ROI4. A decrease of the IR can be associated with recruitment of atelectatic lung regions [16]. In addition to the static ROIs 1–4, consisting of 25% of the lung cross-section each, an adapted approach was performed. Therefore, the effectively ventilated lung regions (IR_adapt_) considering the maximum extent of the entire study (t0, t1, and t2) were split into four equally dimensioned ROIs for each patient individually.

For the calculation of the tidal impedance variation (TIV), at least ten subsequent breathing cycles were selected out of the recorded EIT sequence, and differences of global impedance between end inspiration and end expiration were calculated, averaged, and referred to the baseline measure (which was defined as 100%). Following the use of the RSBI, the ratio between the TIV and the RR was computed for t0, t1, and t2 (RSBI_EIT_).

Changes in end-expiratory lung impedance (ΔEELI) were calculated and set in relation to the baseline measure. The procedure was carried out analogously to the computation of TIV using the same preselected breathing cycles. The EELI values were defined as the local minima of the global impedance curve. ΔEELI may be associated within recruitment/de-recruitment phenomena.

With regard to further analyses of regional ventilation distribution, the regional ventilation delay (RVD) index was used. It describes the delay between the beginning of inspiration and the culmination of a specific impedance threshold and may correlate with regional recruitment within the lung [17]:

$$ R V{D}_i=\frac{\varDelta {t}_i}{t max- tmin}\times 100\%. $$

Although the RVD index was developed for so-called slow inflation maneuvers during MV, in this study it was used for the patients under PSV and under spontaneous breathing in an experimental manner. Even under spontaneous ventilation, we expected meaningful information from the RVD index considering spatial delays during inspiration. For avoidance of confusion, we named this parameter the *regional ventilation delay index during spontaneous breathing* (spRVD).

Moreover, the GI was applied for quantification of ventilation distribution. The ideal value for healthy patients is 0.5. An increasing injury of the lung results in an increase of the GI [18].

$$ G I=\frac{{\displaystyle {\sum}_{x, y\ \in lung}\left| D{I}_{x y}- Median\left( D{I}_{lung}\right)\right|}}{{\displaystyle {\sum}_{x, y\ \in lung} D{I}_{x y}}}. $$

DI = differential impedance, DI_xy_ = pixels in a defined lung region, and DI_lung_ = all pixels representing the lung [18].

### Statistical analysis

Owing to lack of existing data, no power analysis could be performed in advance. Descriptive analysis using means, SDs, and selected quantiles of evaluated parameters was performed separately at t0, t1, and t2. In cases where normal distribution was not given, the median values were analyzed. For comparison of EIT data between the three analysis times, the Friedman test was used, and pairwise comparison between analysis times was performed using the Nemenyi test for multiple comparisons. The logarithmic transformation of the GI measure was used for the analyses.

ROC analysis was performed for testing predictive value of the baseline GI on patients’ ventilatory deterioration after the T-piece trial. A decreased V_T_ of at least 20 ml at the time point t2 in comparison with t0 was defined as a ventilatory deterioration. A subgroup analysis comparing patients with and without decrease in V_T_ was performed. Median values at time points t0 and t2 were analyzed.

## Results

A total of 31 patients with prolonged weaning were included. EIT data collection was feasible in all patients without preterm dropout. Only two patients needed PSV 2 minutes in advance; however, EIT analysis was successfully conducted in all patients. Patient characteristics can be found in Table 1. Most of the patients (64.5%) underwent cardiac surgery in advance. Proportions of 9.6% of patients had cardiac shock and 25.8% had septic shock prior to the prolonged weaning process. All patients underwent long-term treatment (ICU length of stay 13.1 ± 5.4 days) and long-term-ventilation on the ICU (12.4 ± 5.2 days on the ventilator) before admission to the weaning unit.

**Table 1:** Patient characteristics and reasons for intensive care unit admission

Characteristics	Data
Age, years	72.9 ± 7
Male sex, *n* (%)	16 (51.6)
Preexisting COPD	7 (22.5)
CABG surgery, *n* (%)	7 (22.5)
Valve surgery, *n* (%)	3 (9.6)
Combined valve/CABG surgery, *n* (%)	7 (22.5)
Aortic surgery, *n* (%)	3 (9.6)
Septic shock, *n* (%)	8 (25.8)
Cardiogenic shock, *n* (%)	3 (9.6)
ICU length of stay before admission weaning unit, days	13.1 ± 5.4
Ventilator time before admission to weaning unit, days	12.4 ± 5.2

*Abbreviations*: *COPD* Chronic obstructive pulmonary disease, *CABG* Coronary artery bypass graft, *ICU* Intensive care unit

Data are given as number of patients (percent) or as mean ± SD

Hemodynamic data as well as ventilatory and respiratory parameters are displayed in Table 2. A significant decrease of peripheral oxygen saturation was observed on the T-piece without pressure support compared with the other settings (*p* < 0.05).

**Table 2:** Vital data, respiratory data, and blood gas analysis

	t0	t1	t2
Body temperature, °C	37.5 ± 0.5	37.5 ± 0.5	37.5 ± 0.6
HR, beats/minute	89.5 ± 24.9	92.3 ± 26.3	90.8 ± 25.6
SAP, kPa	18.7 ± 3.5	19.2 ± 3.3	18.9 ± 3.5
MAP, kPa	10.9 ± 1.7	11.2 ± 1.9	11.2 ± 2.1
DAP, kPa	8 ± 1.6	8 ± 1.8	8 ± 2.1
Norepinephrine, μg/kg/minute	0.06 ± 0.05	0.06 ± 0.05	0.06 ± 0.05
SpO_2_, %	98 ± 2	95 ± 3^a,b^	97 ± 3
FiO_2_	0.4 ± 0.05	0.4 ± 0.08^c^	0.4 ± 0.05
Pressure support, mbar	9 ± 2.5	NA	9 ± 2
PEEP, mbar	7 ± 1	NA	7 ± 1
Respiratory rate, breaths/minute	23.5 ± 7	26 ± 6	24 ± 6
RSBI, breaths/minute/L	52.2 ± 20.7	NA	51.8 ± 22.4
PaO_2_, kPa	11.3 ± 2.3	9.5 ± 2.0^a,b^	10.9 ± 2.4^a^
PaCO_2_, kPa	5.2 ± 1.1	5.2 ± 1.2	5.1 ± 1.1
pH	7.456 ± 0.04	7.457 ± 0.04	7.461 ± 0.043
HCO_3_^−^, mmol/L	27.7 ± 5	27.5 ± 5	27.3 ± 5
Lactate, mmol/L	0.9 ± 0.3	0.9 ± 0.4	0.9 ± 0.4

*Abbreviations: HR* Heart rate, *SAP* Systolic arterial pressure, *MAP* Mean arterial pressure, *DAP* Diastolic arterial pressure, *SpO*_*2*_ Peripheral oxygen saturation, *FiO*_*2*_ Fraction of inspired oxygen, *PEEP* Positive end-expiratory pressure, *RSBI* Rapid shallow breathing index (rate between respiratory rate and tidal volume), *PaO*_*2*_ Partial pressure of arterial oxygen, *PaCO*_*2*_ Partial pressure of arterial carbon dioxide, *NA* Not applicable, *HCO*_*3*_^*−*^ Bicarbonate

Data are given as mean ± SD

^a^ *p* < 0.05 vs t0

^b^ *p* < 0.05 vs t2

^c^ Calculated FiO_2_ after conversion of oxygen application

**Fig. 2:**
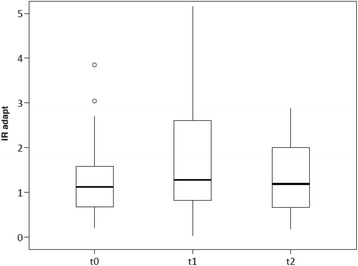
Box plot of impedance ratio analyzed from preset regions of interest ROIs after adapting ROIs on effectively ventilated lung regions (IRadapt) at baseline (t0) as well as during (t1) and after (t2) a spontaneous breathing trial

Fractions of oxygen and ventilatory parameters were comparable at all time points. Patients showed a trend toward a higher respiratory load, which can be seen in slightly higher RRs at the time on the T-piece without pressure support. PaCO_2_ was comparable at all time points. However, in comparison to t0 (*p* < 0.05) and t2 (*p* < 0.05), PaO_2_ was significantly decreased at the time t1 on the T-piece without pressure support. Regarding EIT data, the IR_adapt_ showed no significant differences, except for a trend toward a decreased IR at the time on the T-piece without pressure support (Fig. 2).

TIV was decreased at t1 (72% [interquartile range 45–97%]; *p* = 0.001) and in a similar range as before the SBT at t2 (99% [89–125%]; *p* = 0.375). ΔEELI was −65% [−139% to +1%] (*p* = 0.002) at t1 as compared with t0 and at a similar level as the baseline measure after the SBT at t2 (−1% [−22.5% to 37%]; *p* = 0.689). The EIT-derived RSBI_EIT_ index was significantly increased at t1 as compared with t0 (40.4 [28.2–52.4%] vs. 23.0 [18.5–28.0%]; *p* < 0.001) and t2 (25.8 [18.3–33.1%]; *p* < 0.001), indicating increased rapid shallow breathing at the time on the T-piece.

**Fig. 3:**
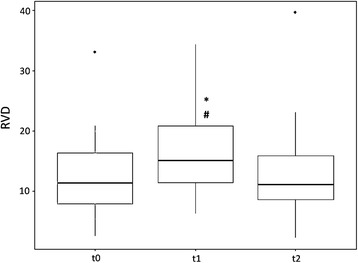
Box plot of regional ventilation delay (RVD) index at baseline (t0) as well as during (t1) and after (t2) a spontaneous breathing trial. ^#^ *p* < 0.05 vs t0; * *p* < 0.05 vs t2

**Fig. 4:**
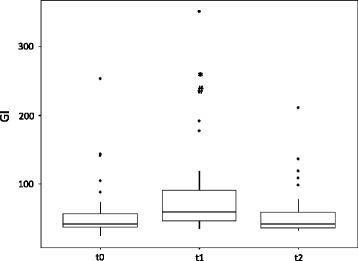
Box plot of global inhomogeneity index (GI) at baseline (t0) as well as during (t1) and after (t2) a spontaneous breathing trial. ^#^ *p* < 0.05 vs t0; * *p* < 0.05 vs t2

Regarding spRVD, a significant increase could be demonstrated at t1. The spRVD was 16.98 ± 7.03 at t1 and significantly decreased toward baseline levels (12.55 ± 6.0, *p* = 0.005) and t2 (12.66 ± 7.1, *p* = 0.002), as illustrated in Fig. 3. For the GI as a marker of increased inhomogeneity within the lung, as depicted in Fig. 4, a significant increase occurred at the time on the T-piece (81.5 ± 62.5) without pressure support compared with t0 (59.3 ± 46.1, *p* < 0.05) and t2 (57.1 ± 39.3, *p* < 0.05).

**Fig. 5:**
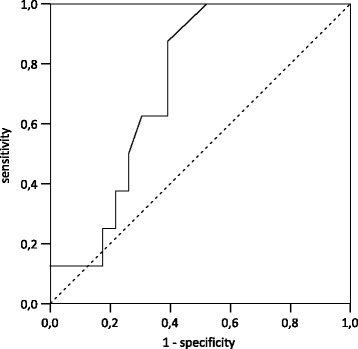
ROC analysis of the global inhomogeneity index on patients with decremental tidal volume after the T-piece trial. AUC 0.73

Thirteen patients showed a ventilatory deterioration with a decreased V_T_ at t2 compared with t0. Assuming a theoretical cutoff value for the baseline GI of >41.5, a sensitivity of 87.5% and a specificity of 60.9% could be shown (AUC 0.73) (Fig. 5).

A subgroup analysis between patients with a decrease in V_T_ and those without a decrease showed no significant changes, except for V_T_ and GI (Table 3). At baseline, V_T_ was higher in the patient group with a decreased V_T_.

**Table 3:** Subgroup analysis of patients with and without decreased tidal volume after spontaneous breathing trials

	SBT with decrease in V_T_		SBT without decrease in V_T_	
	t0	t2	t0	t2
V_T_, ml	**531 [467–627]** ^a^	474 [380–566]	**425 [390–511]** ^a^	480 [425–547]
Respiratory rate, breaths/minute	24 [20–26]	24 [19–31]	22 [17–28]	23 [20–27]
RSBI, breaths/minute/L	44 [38–47]	43 [40–75]	55 [39–64]	46 [36–60]
PaO_2_, kPa	10.9 [9.2–12.4]	10.8 [8.3–11.2]	11.3 [10.0–13.6]	10.5 [9.4–11.9]
PaCO_2_, kPa	4.9 [4.6–5.8]	4.9 [4.4–6.1]	5 [4.5–6.1]	4.7 [4.3–6.1]
IR_global	1.4 [0.4–2.8]	1.3 [0.5–12.5]	0.9 [0.5–1.6]	1.0 [0.5–2.1]
GI	**46 [42–70]**	43 [41–75]	**41 [37–55]** ^a^	40 [35–53]
spRVD	11.9 [7.9–14.9]	13.4 [10.0–16.7]	11.2 [7.7–16.8]	11 [8.4–16.5]

*Abbreviations: V*_*T*_ Tidal volume, *RSBI* Rapid shallow breathing index, *paO*_*2*_ Partial pressure of arterial oxygen, *PaCO*_*2*_ Partial pressure of arterial carbon dioxide, *IR* Impedance ratio, *GI* Global inhomogeneity index, *spRVD* Regional ventilation delay index during spontaneous breathing, *SBT* Spontaneous breathing trial

Measures are stated as median and interquartile range before (t0) and after (t2) spontaneous breathing trial was conducted. A decrease of tidal volume was considered for differences higher than 20 ml to avoid measurement errors

^a^ *p* < 0.05 as compared with the SBT with decrease in tidal volume subpopulation

## Discussion

In the present study, EIT was applied in an observational clinical trial with patients in prolonged weaning. EIT was capable of visualizing and to quantifying effects of spatial ventilation distribution in real time before, after, and during an SBT. Additionally, the GI was able to predict in advance whether a planned SBT would lead to clinical impairment (in terms of a decreased V_T_).

Patients with prolonged weaning are characterized by evident cardiac and pulmonary comorbidities and/or having undergone severe acute critical illness resulting in long-term ventilation and subsequent muscular weakness [19], as well as impaired fluid balance [20, 21]. The main therapeutic goal is a stepwise reduction of ventilatory support (either by gradually reducing the ventilator’s pressure support or by interrupting the ventilator support on a T-piece trial) and a successive transfer of the patients’ respiratory load [22, 23]. However, the first T-piece trials may result in clinical throwback, as seen by delayed clinical symptoms that already show overload of a patient. EIT could be a complementary bedside tool to guide and monitor in a timely way the changes in regional ventilation distribution in patients with prolonged weaning. Therefore, it might contribute to the process of clinical decision making regarding the weaning process. Concretely, EIT enables the clinician to continuously monitor and analyze respiration, even under spontaneous breathing. Among others, a modified RSBI_EIT_ might contribute to the appreciation of whether an SBT should be interrupted prematurely or should even be extended.

Numerous studies have addressed the use of EIT during PSV, but almost exclusively in patients who have received short-term ventilation [11, 24]. In our present study, patients who received long-term ventilation and had a distinctive restriction of respiratory muscles were examined. Calculation of the GI and spRVD yielded a significant increase at the end of a T-piece trial. One explanation could be a decrease in V_T_ as a result of ventilatory compromise during the T-piece trial. Another reason could be decreased lung compliance as a consequence of de-recruitment phenomena. Because measurements of V_T_ during the T-piece trial were not part of this observational study, both potential mechanisms should be taken into consideration.

The RVD index was developed for slow inflation maneuvers during MV [17, 25]. Wrigge et al. used the RVD index during an experimental study of acute lung injury, with slow inflation maneuvers and V_T_ of 12 ml/kg [17]. In another study, Blankman et al. used the RVD index for PEEP settings in patients undergoing controlled MV [26]. However, we believe that transferring the RVD index to spontaneously breathing patients could be meaningful in terms of quantifying temporal and spatial inhomogeneity, even if the underlying effects might be different. Because the spRVD is strongly dependent on the individual patient’s variety, depth, and rate of breathing, a sequence of sober breathing cycles has to be selected, and the assessed absolute measures cannot be compared with those of other patients under MV. Thus, the spRVD seems meaningful for the evaluation of lung heterogeneity in spontaneously breathing patients undergoing a T-piece trial.

The GI describes spatial heterogeneity within the lung, as could be shown in our study. During the T-piece trial, RR was slightly increased and—most probably—V_T_ decreased. At least, the TIV is decreased by about 30%. Moreover, the GI was analyzed for predicting impending clinical impairment of patients in consequence of an SBT. It was found out that in patients with an initial GI >41.5, an SBT should not be performed. By doing so, 87.5% of all SBT failures might be avoided. Although the respective specificity is just 60.9%, this procedure can be applied as a screening method and to support clinical decision making.

From a clinical point of view, a prediction of SBT failure in this particular patient group of prolonged weaning seems meaningful to avoid significant drawbacks. Interestingly, functional parameters of gas exchange or respiratory pattern were not different, as shown in Table 3. This finding may underline the relevance of EIT as a monitoring tool in SBTs, being more accurate than functional parameters of gas exchange.

TIV and ΔEELI are meaningful parameters to monitor success and impending failure, respectively, of the SBT because no ventilator data are available during this period. It could clearly be shown that TIV was reduced during the period of spontaneous breathing as a sign of enhanced respiratory fatigue with a consecutively reduced V_T_. At the same time, EELI was decreased, which might be associated with de-recruitment phenomena that were completely regressive after the SBT.

There is one specific limitation of our study. Within this observation, we examined one patient group that followed a standardized weaning protocol on the T-piece with a scheduled return to PSV. Although our patients showed a tendency toward respiratory fatigue (increase rapid shallow breathing), and although EIT showed significant results, the effects could have been more distinctive if “successful” patients had remained on the T-piece for longer periods. With EIT-derived measures such as the ΔEELI, the TIV, or the RSBI_EIT_, criteria for termination or extension could be defined to guide individual weaning procedures.

Regarding a technical shortcoming of the applied EIT system and its built-in software, it facilitates only qualitative images during examination, whereas quantification demands a complex offline analysis. It would be desirable to develop a dynamic, “easy-to-handle” parameter as a surrogate for regional ventilation distribution. Interestingly, the TREND study group [27] recently published a consensus paper on classifications and structured use as well as systematic analyses of EIT, ending in protocols for the future use of direct EIT parameters and better comparison of clinical observations.

The present study has demonstrated, for the first time to the best of our knowledge, that the EIT could be a useful monitoring tool in patients with prolonged weaning. Further larger studies are needed to investigate the use of EIT in an interventional design.

## Conclusions

Our data demonstrate the safe and reasonable use of EIT in patients with prolonged weaning undergoing T-piece trials. EIT might therefore be a tool to monitor changes in regional ventilation of the lung and heterogeneity. Parameters such as the SBT index by using the ratio of TIV and RR could be complementary to clinical signs of breathing. Quantitative analysis of specific EIT parameters such as the GI might have predictive value regarding clinical deterioration of patients on a T-piece trial.

## Abbreviations

BGA: Blood gas analysis
CABG: Coronary artery bypass graft
COPD: Chronic obstructive pulmonary disease
DAP: Diastolic arterial pressure
DI: Differential impedance
EIT: Electrical impedance tomography
FiO_2_: Fraction of inspired oxygen
GI: Global inhomogeneity index
HR: Heart rate
ICU: Intensive care unit
IR: Impedance ratio
MAP: Mean arterial pressure
MV: Mechanical ventilation
PaCO_2_: Partial pressure of arterial carbon dioxide
PaO_2_: Partial pressure of arterial oxygen
PEEP: Positive end-expiratory pressure
PSV: Pressure support ventilation
ROI: Region of interest
RR: Respiratory rate
RSBI: Rapid shallow breathing index
RVD: Regional ventilation delay index
SAP: Systolic arterial pressure
SBT: Spontaneous breathing trial
SpO2: Peripheral oxygen saturation
spRVD: Regional ventilation delay index during spontaneous breathing
TIV: Tidal impedance variation
V_T_: Tidal volume
ΔEELI: Change in end-expiratory lung impedance
